# Adaptive variations in SARS-CoV-2 spike proteins: effects on distinct virus-cell entry stages

**DOI:** 10.1128/mbio.00171-23

**Published:** 2023-06-29

**Authors:** Enya Qing, Tom Gallagher

**Affiliations:** 1 Department of Microbiology and Immunology, Loyola University Chicago, Maywood, Illinois, USA; NIAID, NIH, Bethesda, Maryland, USA

**Keywords:** coronavirus, SARS-CoV-2, Omicron, virus entry, virus evolution

## Abstract

Evolved SARS-CoV-2 variants of concern (VOCs) spread through human populations in succession. Major virus variations are in the entry-facilitating viral spike (S) proteins; Omicron VOCs have 29–40 S mutations relative to ancestral D614G viruses. The impacts of this Omicron divergence on S protein structure, antigenicity, cell entry pathways, and pathogenicity have been extensively evaluated, yet gaps remain in correlating specific alterations with S protein functions. In this study, we compared the functions of ancestral D614G and Omicron VOCs using cell-free assays that can reveal differences in several distinct steps of the S-directed virus entry process. Relative to ancestral D614G, Omicron BA.1 S proteins were hypersensitized to receptor activation, to conversion into intermediate conformational states, and to membrane fusion-activating proteases. We identified mutations conferring these changes in S protein character by evaluating domain-exchanged D614G/Omicron recombinants in the cell-free assays. Each of the three functional alterations was mapped to specific S protein domains, with the recombinants providing insights on inter-domain interactions that fine-tune S-directed virus entry. Our results provide a structure-function atlas of the S protein variations that may promote the transmissibility and infectivity of current and future SARS-CoV-2 VOCs.

**IMPORTANCE:**

Continuous SARS-CoV-2 adaptations generate increasingly transmissible variants. These succeeding variants show ever-increasing evasion of suppressive antibodies and host factors, as well as increasing invasion of susceptible host cells. Here, we evaluated the adaptations enhancing invasion. We used reductionist cell-free assays to compare the entry steps of ancestral (D614G) and Omicron (BA.1) variants. Relative to D614G, Omicron entry was distinguished by heightened responsiveness to entry-facilitating receptors and proteases and by enhanced formation of intermediate states that execute virus-cell membrane fusion. We found that these Omicron-specific characteristics arose from mutations in specific S protein domains and subdomains. The results reveal the inter-domain networks controlling S protein dynamics and efficiencies of entry steps, and they offer insights on the evolution of SARS-CoV-2 variants that arise and ultimately dominate infections worldwide.

## INTRODUCTION

SARS-CoV-2 has been continuously adapting to human populations from the time of its initial zoonotic emergence in late 2019 (1 - 13). Among the first recognized adaptive changes were the D614G substitutions in the entry-facilitating spike (S) proteins (6, 7, 14 - 17). Subsequent SARS-CoV-2 variants of concern (VOCs) have evolved from these ancestral D614G viruses (8 - 12). Each emergent VOC has had distinctive variations in the S proteins, which have been extensively scrutinized in many research laboratories. Structural studies demonstrated that VOC S proteins have distinctive antigenic epitopes and conformational states (18 - 24), evaluations of virus entry have documented adaptations to host-cell susceptibility factors and cell entry pathways (25 - 29), and animal models of human infection have revealed how S protein variations correlate with viral pathogenicity (14, 30 - 37). Yet even with these many impressive findings, the ways that specific VOC variations alter particular S protein functions during virus-cell entry remain relatively obscure.

**Fig 1:**
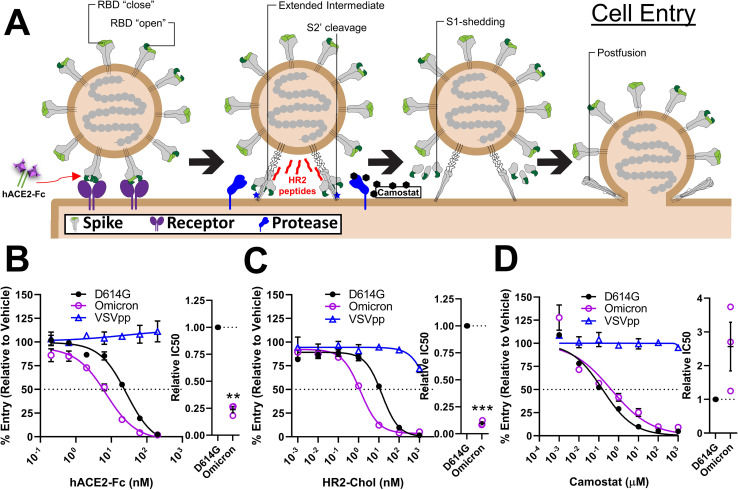
Cell entry assays distinguish D614G and Omicron S protein activities. (A). Schematic of CoV entry process. S functional characteristics including RBD open-close dynamics, extended intermediate formation, S2′ cleavage, and S2 shedding are labeled. hACE2-Fc (testing receptor interactions), HR2 peptide (testing extended intermediate formation), and camostat (testing S2′ cleavage) are indicated at their sites of action. (B through D) SARS-CoV-2 virus-like particle RNA transductions were used to evaluate Vero-E6-hACE2^+^-hTMPRSS2^+^ cell entry processes. Transductions were measured in the presence of hACE2-Fc (B), HR2 peptides (C), and camostat (D). VSVpp transductions were included as negative controls. Data were normalized to vehicle controls. *Left*, dose-response curves between treatment and % entry. *Right*, experiments on the *left* were repeated three times (*N* = 3), and the doses generating 50% inhibition (IC50) were plotted. Mean and standard error of the mean (SEM) are depicted. Deviations from the reference value of 1.0 (D614G S) were analyzed by one-sample *t* tests. **, *P* < 0.01; ***, *P* < 0.001.

SARS-CoV-2 S proteins are complex homotrimeric integral-membrane glycoproteins (38 - 40). Each monomer comprised several domains weaving together to form the dynamic molecular machines directing cell entry [(41 - 43) and Fig. 1A]. Trimeric S protein ectodomains contain a distal S1 cap and virion-proximal S2 stalk. S1 comprises four domains (38 - 40): N-terminal domain (NTD), receptor-binding domain (RBD), subdomain 1 (SD1), and subdomain 2 (SD2). In cell entry, flexible RBD petals “open” to expose hACE2 receptor-binding sites (44, 45), while NTDs and SD1/SD2 execute control in this essential RBD opening process (15, 18, 46, 47). Receptor binding induces S protein conformational changes (44, 45, 47 - 50) that include exposure of highly conserved proteolytic substrate sites (47, 50 - 52). Cleavage at these S2′ sites triggers S-directed membrane fusion. Membrane fusion requires major structural transitions in the S2 stalks, which unfold into extended intermediate structures that bridge viral and cellular membranes and then pull opposing membranes together by refolding into helical bundles (40, 53 - 55). This completes virus entry (see Fig. 1A). All of these steps in virus entry are thoroughly documented, yet the ever-evolving SARS-CoV-2 VOCs bring up important new questions. It remains unclear how VOC mutations refine specific entry steps, in ways that might adapt SARS-CoV-2 for continued maintenance in the human population.

New VOCs emerging from ancestral D614G viruses outcompete predecessors. At present, the Omicron group of VOCs is dominating. Omicron BA.1 contains three to six times more S variation than preceding VOCs (10, 13). Omicron viruses show significant antigenic drift (23, 28, 29, 56), indicating selective pressures imposed by neutralizing antibodies (32, 57 - 60). Other selective forces are also evident. Omicron BA.1 S structural studies revealed altered receptor affinity, RBD dynamics, and S2′ accessibility (23, 29, 61 - 64). Cell culture experiments indicated a shift toward endosomal entry routes (27, 28, 65 - 68). *Ex vivo* airway cell and *in vivo* animal infections showed that Omicron growth is adapted to nasal epithelia (27, 37, 65, 69 - 73). These observations make it clear that selective forces distinct from neutralizing antibodies drive SARS-CoV-2 variations. Identifying these selective forces and determining how they refine S protein entry functions will help to explain SARS-CoV-2 evolutionary trajectories as this virus becomes endemic in humans.

In this study, we used reductionistic *in vitro* systems to probe SARS-CoV-2 S proteins as they execute specific entry steps. We compared ancestral D614 to Omicron virus particles and found that Omicron (BA.1) was set apart by its hyperactive response to hACE2 receptors and membrane fusion-activating proteases. Omicron BA.1 also showed distinctive sensitivity to peptide-based membrane fusion inhibitors. In discerning the specific mutations causing these hypersensitivities, we identified new inter-domain interactions that control S protein cell entry functions.

## RESULTS

### Functional distinctions between ancestral and Omicron SARS-CoV-2 S proteins

We utilized a SARS-CoV-2 virus-like particle (VLP)-based RNA transduction system (74 - 76) to compare the cell entry functions of variant SARS-CoV-2 S proteins. With this system, we analyzed several changes in S proteins that take place during entry, including receptor-induced conformational changes and fusion-activating proteolytic cleavages (Fig. 1A). Receptor reactivities were assessed by titrating inhibitory soluble receptors (hACE2-Fc) into inoculating VLPs. Here, Omicron (BA.1) VLPs were about four times more sensitive to receptor-mediated neutralization than ancestral D614G VLPs (Fig. 1B). The extended S conformations that are poised for membrane fusion were probed by titrating inhibitory HR2 peptides that bind specifically to these structures and prevent subsequent transitions to postfusion forms (53) (see Fig. 1A). Here, Omicron VLPs were about 10 times more sensitive to neutralization than D614G VLPs (Fig. 1C). Finally, VLP responses to fusion-activating proteases were evaluated by titrating camostat, a protease inhibitor specific for the type II serine proteases that cleave receptor-bound CoV S proteins (27, 28, 77, 78). Relative to D614G VLPs, the Omicron VLP transductions were about two times less sensitive to inhibitory camostat (Fig. 1D), suggesting that Omicron is hyper-responsive to protease-activated cell entry.

**Fig 2:**
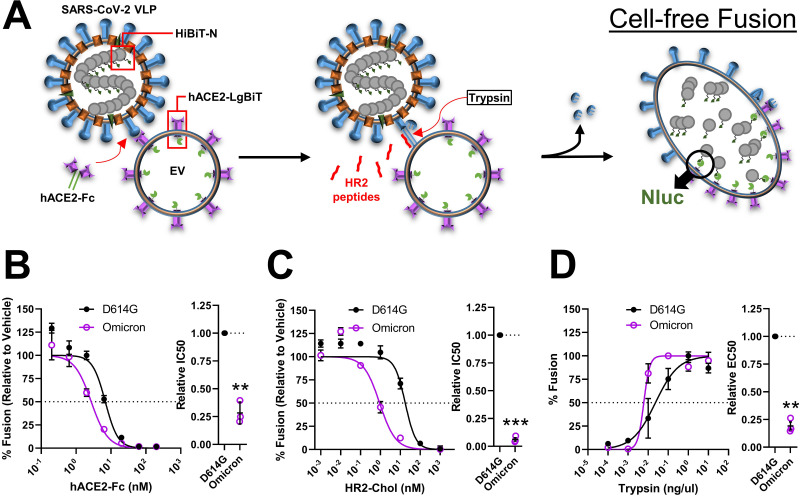
Cell-free fusion assays distinguish D614G and Omicron S protein activities. (A) Schematic of the cell-free fusion system. VLP-EV membrane fusion was used to assess hACE2-Fc (testing receptor interactions), HR2 peptide (testing extended intermediate formation), and trypsin (testing S2′ cleavage) sensitivities. Membrane fusion was measured by fusion-dependent Nluc formation. (B–D) Results comparing D614G and Omicron S-bearing VLP fusions in the presence of hACE2-Fc (B), HR2 peptides (C), and trypsin (D). *Left*, dose-response curves between treatment and % fusion. *Right*, experiments on the *left* were repeated three times (*N* = 3), and the doses generating 50% inhibition (IC50) or 50% of maximal fusion (EC50) were plotted. Mean and SEM are depicted. Deviations from the reference value of 1.0 (D614G S) were analyzed by one-sample *t* tests. **, *P* < 0.01; ***, *P* < 0.001.

We advanced the comparisons of D614G and Omicron VLPs by utilizing *in vitro* fusion assays (52, 79). These assays faithfully mimic authentic virus-cell entry (Fig. 2A). They use SARS-CoV-2 VLPs that are engineered to contain nanoluciferase (Nluc) “HiBiT” fragments. “HiBiT” VLPs are incubated with hACE2-positive extracellular vesicles (EVs) that contain internal Nluc “LgBiT” fragments. Protease-triggered VLP-EV membrane fusions then allow HiBiT and LgBiT to come together, generating Nluc activities that are measured as readouts for S protein-mediated receptor interaction and membrane fusion. This system permits a cell-free, exogenous protease-dependent dissection of the steps required to initiate infection (47, 52, 53, 79, 80). Results from the cell-free assays confirmed that Omicron VLPs were hypersensitive to neutralization by soluble ACE2 (Fig. 2B) and to fusion-inhibitory HR2 peptides (Fig. 2C). The results also demonstrated that the Omicron VLPs were hypersensitive to activation by trypsin (Fig. 2D); trypsin is a serine protease that mimics the TMPRSS2 that cleaves and activates fusion in several coronaviruses (43, 49, 81 - 88). Coherent data acquired from the VLP-cell transduction and VLP-EV fusion assays suggest that Omicron viruses have evolved from ancestral D614G to become more responsive to receptor-induced conformational changes, more prone to durably display fusion-readied conformational intermediates, and more sensitive to fusion-activating proteases en route to virus-cell membrane fusion and productive cell entry.

### S protein domains controlling neutralization

**Fig 3:**
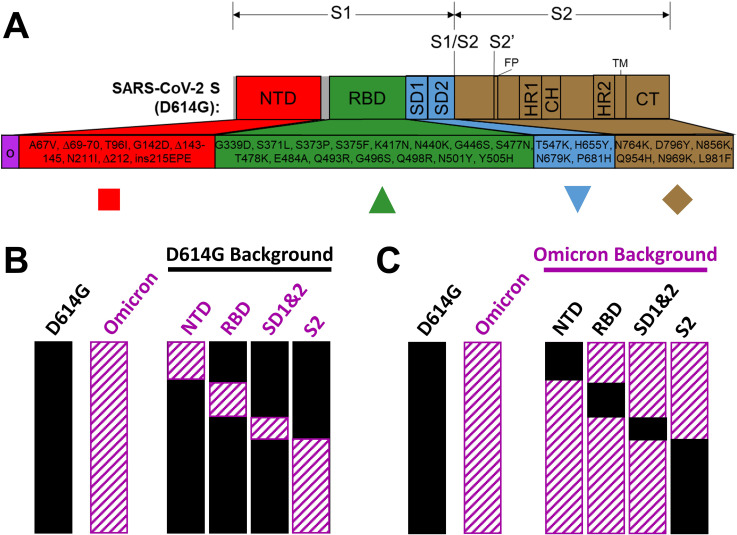
Schematics depicting recombinant S proteins. (A) Linear representation of SARS-CoV-2 S domains. Omicron (O) BA.1 changes relative to ancestral D614G S are indicated. Recombined domains are color coded and designated with specific symbols that are used consistently in Fig. 4 to 7. (B) Depiction of Omicron S domains (striated) in D614G backgrounds. (C) Depiction of D614G S domains (solid) in Omicron backgrounds.

**Fig 4:**
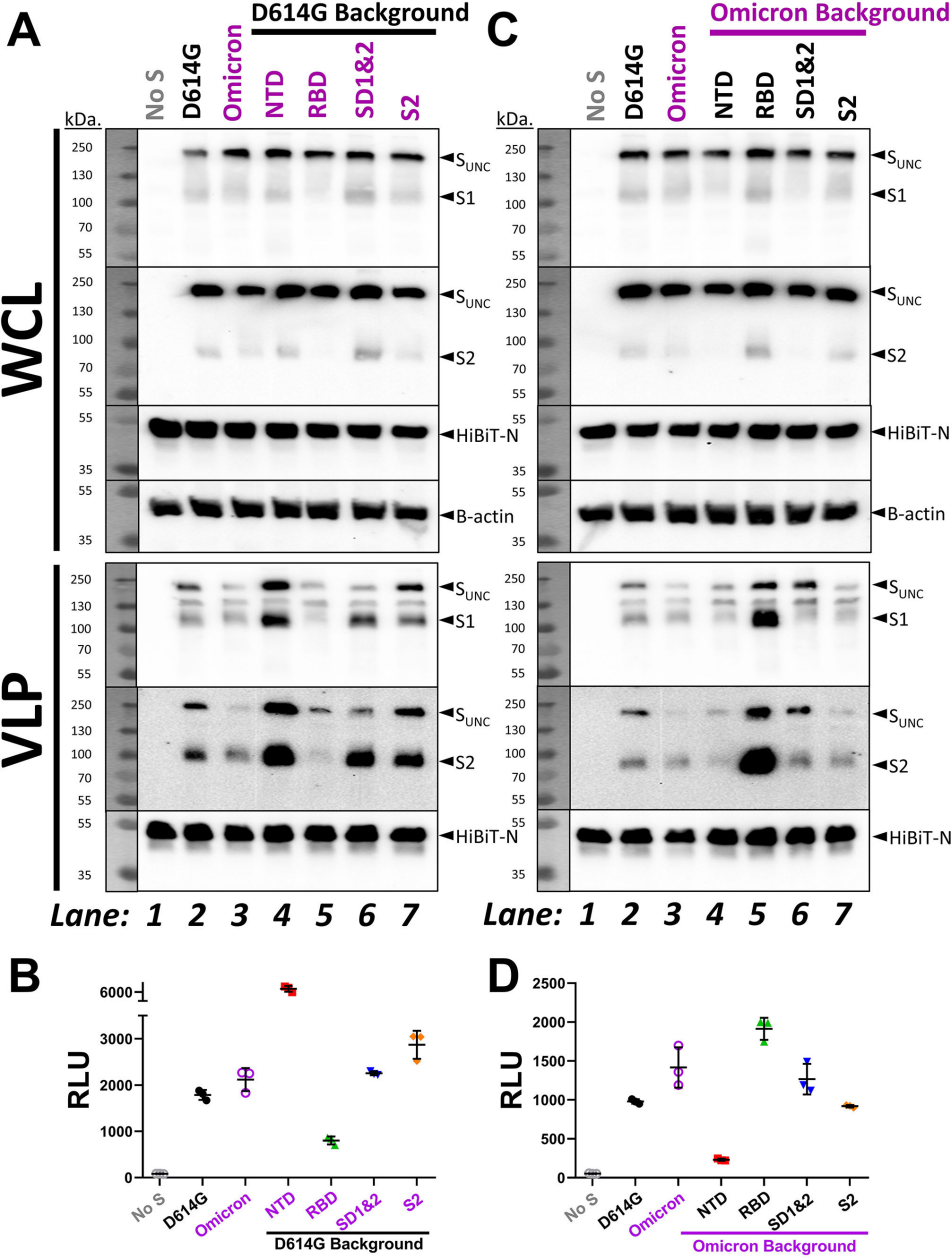
Detection of recombinant VLP S proteins and fusion potentials. (A and C) Western blot analyses of recombinant S proteins in VLP-producing WCL (top) and purified VLPs (bottom). Molecular weights (kDa) are indicated at left; uncleaved S (S_UNC_), S1, S2, HiBiT-N, and β-actin are labeled at right. (B and D) Dot plots of Nluc relative light units (RLU) measured from cell-free fusion assays of the indicated recombinant VLPs.

Omicron variants have acquired deletion and substitution mutations in several S protein domains. The mutations in Omicron BA.1 are depicted in Fig. 3A; of note, the currently dominant Omicron XBB.1.5 shares 23 of these mutations, with notable BA.1/XBB.1.5 hypervariability in NTDs (89). We anticipated that the Omicron mutations in RBDs would confer susceptibility to RBD-binding hACE2. Similarly, we expected that Omicron mutations in S2 would increase sensitivity to S2-binding HR2 peptide neutralization. To address these speculations, we engineered recombinant VLPs in which domains were exchanged between Omicron and ancestral D614G and then measured their relative sensitivities to neutralization. We replaced D614G domains with those from Omicron (Fig. 3B, “D614G background”) and reciprocally replaced Omicron domains with those from D614G (Fig. 3C, “Omicron background”). VLP production was then evaluated. In VLP-producing cells, all parental and recombinant S proteins were produced equivalently, with most cellular S being uncleaved (Fig. 4AC, WCL, note S_UNC_ relative to HiBIT-N in Whole Cell Lysates). In all WCL samples, only small proportions of cleaved S1 and S2 products had accumulated to variable extents. S proteins on purified VLPs contained more cleaved S1/S2 (Fig. 4AC, VLP), as expected for S proteins transiting through furin-containing exocytic organelles. S protein levels among the VLPs varied. Most notably, Omicron NTD mutations increased D614G S incorporation into VLPs (Fig. 4A, lane 4), while Omicron RBD mutations decreased S incorporation (Fig. 4A, lane 5). Conversely, reverting Omicron NTD mutations back to D614G decreased Omicron S incorporation (Fig. 4C, lane 4), while reverting the Omicron RBD mutations markedly increased incorporation (Fig. 4C, lane 5). These findings are consistent with a complex co-evolution of NTD and RBD domains, with selective forces presumably at the level of S protein folding, endoplasmic reticulum (ER)-Golgi transport to virion budding sites, or assembly into virions.

**Fig 5:**
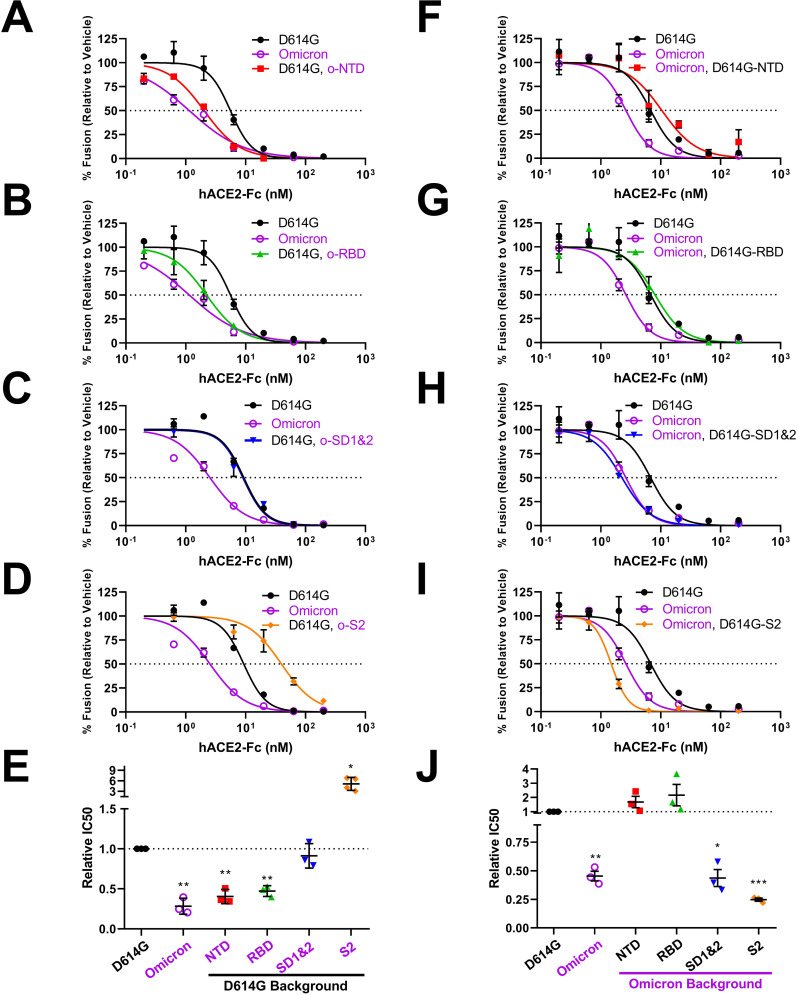
Neutralization of recombinant VLPs by hACE2-Fc. (A–D) hACE2-Fc neutralization profiles for D614G VLPs with Omicron domain replacements. (E) Experiments were repeated at least three times, and 50% inhibition values (IC50) were plotted. (F–I) hACE2-Fc neutralization profiles of Omicron VLPs with D614G domain replacements. (J) Experiments were repeated at least three times, and 50% inhibition values (IC50) were plotted. Mean and SEM are depicted. Deviations from the reference value of 1.0 (D614G S) were analyzed by one-sample *t* tests. *, *P* < 0.05; **, *P* < 0.01; ***, *P* < 0.001.

VLPs contained HiBiT-N and were further evaluated in cell-free fusion assays (Fig. 2A). Parental D614G and Omicron VLPs generated comparable maximum fusion levels (Fig. 4B and D), consistent with their similar S protein levels. Omicron NTD mutations increased D614G-based VLP fusion levels (Fig. 4B, lane 4), while Omicron RBD mutations decreased the fusion levels (Fig. 4B, lane 5). Conversely, reverting Omicron NTD mutations back to D614G decreased Omicron-based VLP fusions (Fig. 4D, lane 4), while RBD reversions increased fusion (Fig. 4D, lane 5). Therefore, the maximum fusion levels elicited by VLPs varied in accord with VLP-associated S protein levels.

### S protein domains controlling susceptibility to receptor-mediated neutralization

Next, we introduced hACE2-Fc into the cell-free fusion assays to assess soluble receptor neutralization. For those recombinant VLPs in the D614G backgrounds (Fig. 5A through E), we found that Omicron RBD mutations increased hACE2-Fc neutralization by about threefold (see Fig. 5B, and Fig. 5E for relative IC50 data). This result was in-line with expectations (29, 62 - 64). However, Omicron NTD mutations also increased neutralization to similar extents (Fig. 5A and E). Omicron SD1 and 2 replacements effected no changes to the D614G-characteristic neutralization (Fig. 5C and E). Omicron S2 mutations significantly decreased neutralization sensitivity (Fig. 5D and E). Clearly, the S protein responses to soluble receptors involve more than the RBDs.

For those recombinant VLPs in the Omicron background (Fig. 5F through J), we found that reverting Omicron NTD or RBD mutations to D614G reduced Omicron VLP neutralization back to the D614G levels (see Fig. 5F and G, and Fig. 5J for relative IC50 data). These results concorded well with those made in the D614G backgrounds. The return to D614G SD1 and 2 had no effects on Omicron neutralization (Fig. 5H and J). However, return to D614G S2 slightly increased neutralization sensitivity (Fig. 5I and J). Overall, these results reveal a complex and broad range (±~sixfold) control of SARS-CoV-2: receptor interactions that extend beyond the RBDs, with S2 changes having a significant role in effecting responses to soluble receptors.

### S protein domains controlling susceptibility to fusion-inhibitory peptides

**Fig 6:**
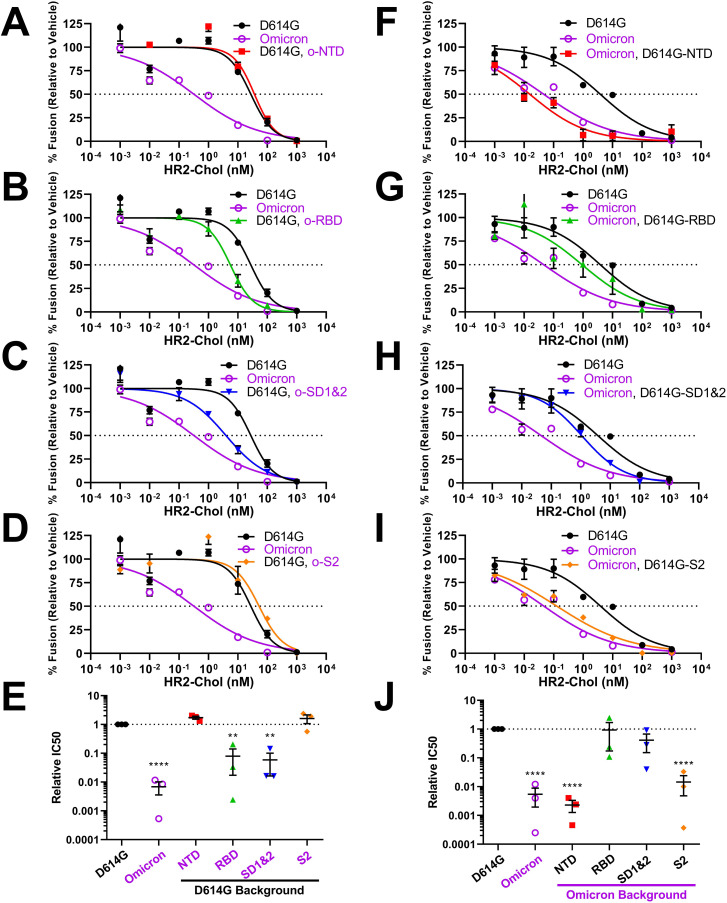
Neutralization of recombinant VLPs by fusion-inhibitory HR2 peptides. (A–D) HR2 peptide neutralization profiles for D614G VLPs with Omicron domain replacements. (E) Experiments were repeated three times, and 50% inhibition values (IC50) were plotted. (F–I) HR2 peptide neutralization profiles for Omicron VLPs with D614G domain replacements. (J) Experiments were repeated three times, and 50% inhibition values (IC50) were plotted. Mean and SEM are depicted. Deviations from the reference value of 1.0 (D614G S) were analyzed by one-sample *t* tests. **, *P* < 0.01; ****, *P* < 0.0001.

We next identified mutations that sensitize Omicron VLPs to HR2 peptide neutralization. Quite unexpectedly, we found that mutations in S2 did not effect any changes to the characteristic D614G and Omicron VLP neutralization titers (Fig. 6D, E, I, and J). Similarly, NTD mutations did not change parental neutralization titers (Fig. 6A, E, F, and J). It was RBD and SD1 and 2 mutations that altered VLP neutralization sensitivities, to levels between those of D614G and Omicron (Fig. 6B, C, E, G, H, and J). Thus the evolution of RBDs and SDs 1 and 2 exerts inter-domain effects that extend to the fusion-inducing S2 domains, generating an S protein refolding pathway that leaves extended intermediates durably sensitized to neutralization by HR2 peptides.

### S protein domains controlling susceptibility to fusion-activating proteases

**Fig 7:**
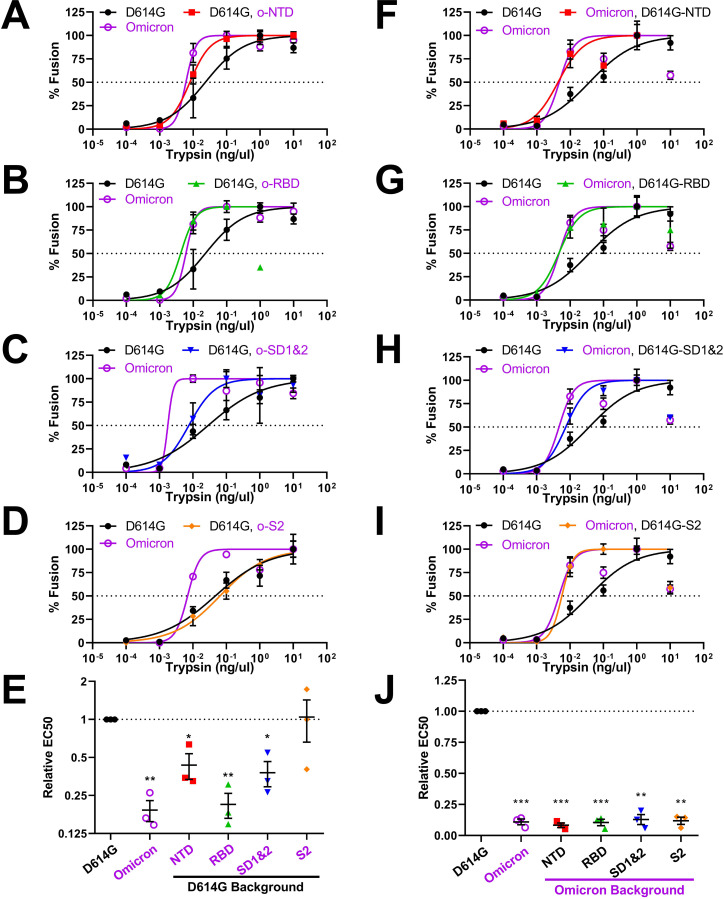
Activation of recombinant VLPs by trypsin proteases. (A–D) Trypsin activation profiles for D614G VLPs with Omicron domain replacements. (E) Experiments were repeated three times, and 50% effective dose values (EC50) were plotted. (F–I) Trypsin activation profiles for Omicron VLPs with D614G domain replacements. (J) Experiments were repeated three times, and 50% effective dose values (EC50) were plotted. Mean and SEM are depicted. Deviations from the reference value of 1.0 (D614G S) were analyzed by one-sample *t* tests. *, *P* < 0.05; **, *P* < 0.01; ***, *P* < 0.001.

We next dissected the domain-specific mutations sensitizing Omicron VLPs to proteolytic actation of membrane fusion. Here, we found that each Omicron S domain could be reverted back to D614G without reducing the characteristic Omicron hypersensitivity to protease activation (Fig. 7F through J). This result suggested that substitutions in several domains work together to control viral responses to proteolytic activation. This notion was addressed by evaluating the converse installation of Omicron domain mutations into the D614G backgrounds. Here, the Omicron S2 domains did not change D614G-specific protease susceptibilities (Fig. 7D and E), making it clear that S2 mutations are not exerting effects on this key property of the S proteins. NTD and SD1 and 2 domains partially contributed to heightened susceptibility (Fig. 7A, C, and E), while RBD changes completely instilled Omicron-level protease sensitivity (Fig. 7B and E). While further insights into the structure and function of some of these complex inter-domain interactions are needed, these current results demonstrate that NTDs and RBDs, along with their associated subdomains 1 and 2, all operate to control proteolytic activation of membrane fusion. The intricacy of this control is evident from the non-reciprocal nature of the domain exchanges.

Of note, VLP sensitivities to soluble receptors, fusion-inhibitory peptides, and fusion-activating proteases did not correlate in any way with VLP S protein densities, S1/S2 cleavage extents, or maximal VLP fusion potentials (see Fig. S1 and S2). For example, NTD and RBD domain exchanges have significant effects on VLP-associated S levels (Fig. 4, compare lanes 4 and 5), but have no effects on sACE2-Fc neutralization (Fig. 5). Therefore, the recombinant S proteins have distinct sensitivities to inhibitors and activators that are independent of VLP S densities or S1/S2 cleavage status.

### Specific S2 amino acid substitutions controlling VLP susceptibilities to receptor-mediated neutralization

We next focused attention on specific amino acid substitutions in S2 that control the distinctive character of Omicron VLPs. This concentration on S2 developed from our findings that adaptations in this region altered virus responses to ACE2 receptors (Fig. 5) but not to peptides targeting extended intermediates (Fig. 6) or to proteases (Fig. 7). We considered these results intriguing and unexpected because S2 domains do not directly engage ACE2 receptors yet they do directly bind HR2 fusion-inhibitory peptides and they do contain the S2′ substrate sites that are cleaved by activating proteases. Furthermore, the functional significance of S2 domain evolution is currently limited and far less understood than the thoroughly evaluated RBDs (23, 27 - 56 - 61 - 63 - 69 - 70 - 69 - 70).

**Fig 8:**
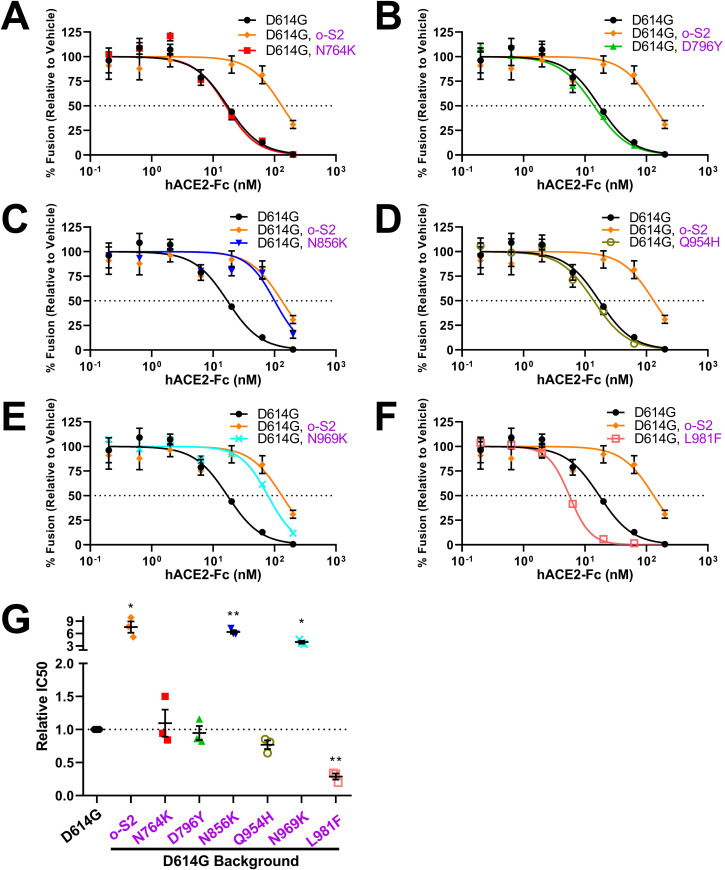
Identification of specific S2 mutations controlling responses to hACE2-Fc. (A–F) Omicron S2 mutations were introduced into D614G VLPs. hACE2-Fc neutralization profiles for the mutant VLPs were obtained in parallel with parental D614G and recombinant D614G, Omicron (o-)S2 VLPs. (G) Experiments were repeated three times, and 50% inhibition values (IC50) were plotted. Mean and SEM are depicted. Deviations from the reference value of 1.0 (D614G S) were analyzed by one-sample *t* tests. *, *P* < 0.05; **, *P* < 0.01.

The S2 residues exchanged in the VLPs include D614G-to-Omicron N764K, D796Y, N856K, Q954H, N969K, and L981F (Fig. S3). We generated D614G VLPs with single residue S2 substitutions and then evaluated sACE2-Fc neutralization (Fig. 8). Three of the substitutions (N764K, D796Y, and Q954H) had no effect on D614G-characteristic neutralization (see Fig. 8G for relative IC50 data). Two substitutions (N856K and N969K) generated resistance to sACE2-Fc, and one (L981F) had the opposite effect of hypersensitizing VLPs to sACE2-Fc (Fig. 8G). These findings bring out three S2 residues, N856, N969, and L981, as side chains controlling RBD dynamics and subsequent VLP inactivation by soluble receptor ligations. The findings highlight the evolution of inter-domain communications in modifying S protein functions.

## DISCUSSION

### Worldwide dominance of Omicron-like viruses

Adaptive variations in S proteins promote continuous human SARS-CoV-2 infection and transmission. SARS-CoV-2 S protein structures (23, 29, 38 - 46 - 55 - 61 - 90 - 91 - 91), antigenicities (23, 28, 29, 56), and interactions with host susceptibility factors (26 - 31 - 33 - 65 - 92 - 93 - 93) have been extensively evaluated in relation to viral pathogenicity (94 - 97). Here, we add to the impressive knowledge of sarbecovirus evolution by comparing ancestral viruses with more recently emerged Omicron variants. The comparisons were made using *in vitro* assays that measure receptor interactions and membrane fusion-inducing properties of S proteins present on VLPs. The results showed that Omicron (BA.1) S proteins were set apart by their hypersensitivity to hACE2 receptors and to fusion-activating proteases. Coronavirus S proteins respond to receptor binding and proteolytic scission by undergoing dramatic conformational changes (40, 45, 49, 85, 87). The findings are consistent with enhanced structural dynamics in the Omicron S proteins, with conformationally “open” states a distinctive feature of the Omicron S proteins as they executed cell entry steps. We suggest that these evolved attributes of the Omicron S proteins contribute to the current worldwide dominance of Omicron-like viruses.

### Concordant findings in the field

Most results in this manuscript come from highly reductionist cell-free assay platforms. These assays permit fine distinction of several steps in virus-cell entry. They can be used to reveal S protein conformational intermediates during cell entry (52, 53). They can measure S-mediated membrane fusion under well-defined reaction conditions (47, 52, 53, 80). Notably, the results of these cell-free assays cohered with those obtained using VLP-cell entry assays (Fig. 1). They also appear to align with those obtained from natural Omicron virus infection models. Omicron VOCs outcompete ancestral viruses in nasal epithelial cultures, with greater than 50-fold increased titers (37, 73). Omicron VOCs effectively utilize TMPRSS2 in *ex vivo* and *in vivo* host environments (65, 98), in accordance with our results showing that the related serine protease trypsin activates Omicron VLP membrane fusion potential at neutral pH. *In vivo* infections by Omicron VOCs are preferentially suppressed by fusion-inhibitory HR2 peptides (99), as they are in VLP fusion assays (Fig. 6). The cell-free fusion assays, therefore, are one dependable addition to an arsenal of assays that are needed to fully characterize SARS-CoV-2 and other CoVs as they evolve alternative entry functions.

### Correlating Omicron-like character to specific adaptive variations

Here, we made genotype-phenotype correlations by comparing a series of recombinant VLPs for Omicron-like character. S protein domain recombinations arise naturally, and recombinant CoVs are often selectively amplified in nature (100 - 107). Previous engineered domain swaps in coronavirus S proteins have brought insights on structure-function relationships (108 - 113). These foundational reports highlighted the roles of specific domains on viral entry, tropism, and pathogenesis. Our study fits in among these precedents and puts a special focus on unraveling inter-domain interactions in directing S protein functions.

The results herein also bring Omicron VOC characteristics into sharper focus. Several recent studies implicating individual SARS-CoV-2 variations with Omicron-like character have provided insights on the mutations affecting entry routes, specifically the preferential trafficking of Omicron viruses into endosomes before cathepsin-triggered membrane fusion (27, 28, 65 - 115 - 114 - 115). Yet it has been shown that Omicron *in vivo* infections require cell-surface TMPRSS2 for fusion triggering (65, 98). Therefore, the physiologically relevant entry routes taken by VOCs are under active investigation, and it remains unclear how differential entry processes might explain the superior fitness of Omicron VOCs in human transmission and infection. We did not evaluate VLP-cell entry routes in this study, rather we focused on cell-free assays of S functions to elucidate Omicron behaviors that might relate to human adaptation. Our results revealed inter-domain networks controlling specific S functions, as discussed in the following paragraphs.

### Inter-domain communications controlling virus-receptor interactions

Receptor binding generates conformational changes in CoV S proteins (44, 45, 47, 50, 51, 87, 116). These changes proceed to cell membrane fusion and infection when viruses are juxtaposed to host cell surfaces. However, when viruses are not on cell surfaces, soluble receptors drive the conformational changes through to permanent inactivation, i.e., neutralization (49, 52, 87, 117). We contend that Omicron hypersensitivity to hACE2-Fc neutralization reflects highly reactive and ultimately inactivating receptor-induced conformational transitions in the S proteins. One might expect that the Omicron variations controlling this hypersensitivity would be within the RBDs, as it is known that Omicron RBDs have relatively high affinities for ACE2 (27, 29, 63, 64). Yet we found controlling variations in RBD, NTD, and S2 domains, indicating multidomain controls (Fig. 5). These findings are in-line with recent reports. It is known that the NTDs can control RBD dynamics (18, 47). As for S2, there are few precedents. A single-molecule Förster resonance energy transfer (smFRET) study revealed that an S2-specific antibody, 1A9, bound at the base of S trimers and promoted distal RBD opening (118). Furthermore, a particular Omicron S2 mutation, N856K, moved RBDs toward S protein threefold axes, through connections between D568 and T572 on SD2 (23, 29). We identified the N856K change as a central mediator of sACE2 sensitivity. In addition to N856K, we also identified two other Omicron S2 changes that control responses to sACE2, N969K, and L981F (Fig. 8). Unlike N856K, structural studies did not reveal mechanistic insights connecting these residues to RBD dynamics (23, 29). Elucidating how N969K and L981F control RBD conformations will further our understanding of CoV S2 adaptations.

### Inter-domain communications controlling the membrane fusion stage

At the later CoV entry stages, unstable extended intermediate conformations collapse into helical bundles to complete virus-cell membrane fusion (40, 53, 85, 87, 119 - 122). This collapse, and the resulting membrane fusion process, is prevented by the HR2 peptides that bind only to the intermediate conformations (34, 53, 99, 122 - 124). Omicron viruses were most effectively blocked by HR2 peptides (Fig. 2C). This implies prolonged existence of the Omicron intermediate conformations, i.e., there may be faster formation and/or slower collapse of intermediates, leaving Omicron entry durably sensitized to HR2 binding and neutralization. The rate of extended intermediate formation is conveyed by S1 domains, while the rate of collapse may be a complex function of the time it takes for S2 domains to congregate and cooperatively “pull” viral and cellular membranes into proximity (125, 126). We unexpectedly found that Omicron mutations in S1 RBD and SD made VLPs hypersensitive to HR2 peptide neutralization, while mutations in S2 had no effect (Fig. 6). This suggests that Omicron has adapted a rapid formation of extended intermediates following ACE2:S1 binding. This proposition is consistent with Omicron hypersensitivity to hACE2 engagement and to proteolytic scission at S2′. However, we do not exclude the possibility that Omicron is slow to refold extended intermediates into postfusion bundles. The kinetics of intermediate collapse requires additional scrutiny, especially in light of reports that coronaviruses may “time” the rates at which intermediates refold through changes in S2 domains (122). Of note, a slow conversion to postfusion states would be consistent with Omicron viruses frequently trafficking into endosomes before completing the cell entry process (27, 28, 65 - 68).

### Inter-domain communications controlling proteolytic activation

After receptor engagement, CoV S proteins are cleaved by a variety of host proteases at or near a region termed S2′ (47, 49, 51, 52, 84). Using trypsin as the host protease, we previously demonstrated that VLP membrane fusion directly correlates with cleavage at S2′ (52). In the present study, we found that Omicron VLPs were particularly sensitive to fusion-activating S2′ cleavage by trypsin. This hypersensitivity is traced to Omicron changes in each of several S1 domains; NTD, RBD, and SD1 and 2 (Fig. 7). Our results are in-line with the previous finding that NTDs from each VOC differentially enhanced protease sensitivity (52). With respect to S2 mutations, we note that engineered substitutions at inter-protomer contacts will increase S2′ cleavages (52); however, the set of natural Omicron S2 mutations did not operate similarly (Fig. 7). Structural and biochemical results show that the Omicron BA.1 S2′ site is more transiently exposed than D614G S (23, 50, 61). Our data demonstrate that S2′ exposure and proteolytic sensitivity can be controlled by mutations in several S protein domains, implying multiple evolutionary pathways to SARS-CoV-2 adaptation for host proteases. Additional research is necessary to discern the exact S structural intermediates exposing S2′ for proteolysis.

### Selective forces driving sarbecovirus variation

**Fig 9:**
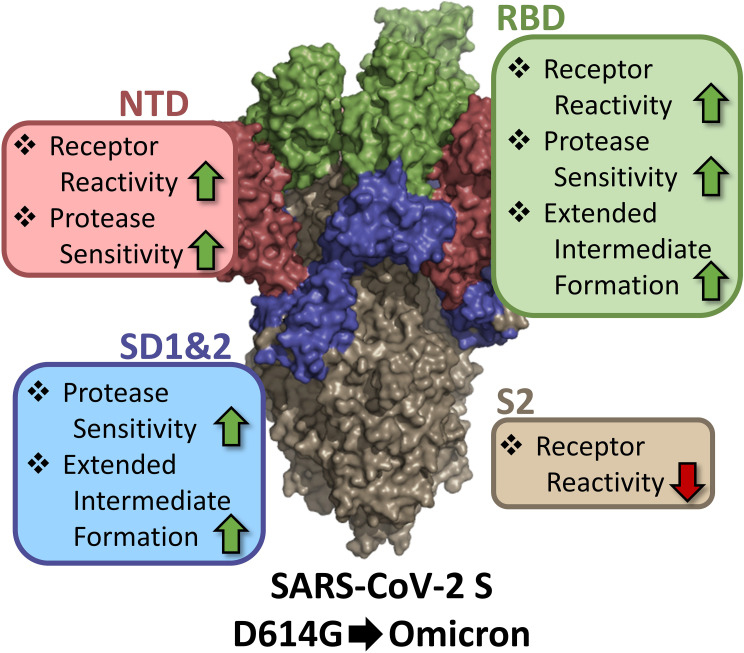
Domain contributions to specific SARS-CoV-2 S functions. NTD (red), RBD (green), SD1 and 2 (blue), and S2 (beige) are color coded onto the surface representation of SARS-CoV-2 S (PDB: 7KRR). Enhancements are indicated by upward green arrows, and reductions are indicated by downward red arrows.

While antibodies clearly drive changes conferring neutralization escape (32, 57 - 60), several other distinct selective pressures impinge upon sarbecovirus and other CoV S proteins. Several variations in SARS-CoV-2 SD1 and 2 and S2 are not within any known neutralizing antibody epitopes (23, 50, 116, 127 - 131), and the fact that these alterations reset responses to host receptors and proteases are consistent with these entry factors driving evolutionary pathways. The entry-altering functions of the SD and S2 changes also begin to reveal the diversity of inter-domain communications that optimize SARS-CoV-2 infection in disparate host environments. Variations adjusting cell entry were found throughout S domains, many with no known roles in direct binding to host factors. Specifically, receptor reactivity was controlled by NTD, RBD, and S2, protease sensitivity by NTD, RBD, and SD1 and 2, and abundance of the extended intermediate by RBD and SD1 and 2 (Fig. 9). Mutations in several S domains, each resetting virus responsiveness to the same host entry factors, highlight how CoVs maximize fitness by finely tuning cell entry processes. Complex bidirectional tuning controls are evident in the Omicron viruses; as an example, Omicron NTD and RBD mutations increase responses to hACE2 receptors while Omicron S2 mutations decrease the same responses (Fig. 5). Inter-domain communications provide SARS-CoV-2 and other CoVs with evolutionary flexibilities and rapid adaptations to zoonotic environments.

Emergence and subsequent dominance of Omicron VOCs is most often explained by its resistance to neutralization by ancestral virus infection and vaccine-induced antibodies (27, 28, 37, 56, 62, 76, 132). Yet even in the complete absence of antiviral antibodies, Omicron fitness is superior to ancestral variants—Omicron clearly outcompetes prior VOCs in *ex vivo* explants of human nasal epithelia (37, 65). Of note, Omicron VOCs do not gain advantage in several other *ex vivo* and *in vivo* animal models (37), and this confounds efforts to explain VOC adaptive mechanisms. Hence the reductionist *in vitro* fusion assays employed in this study might be highlighted, as they did reveal Omicron VOC characteristics that are consistent with its heightened ability to infect the upper airways. Elevated reactivity to hACE2 receptor binding, facile formation of extended intermediate conformations, and hypersensitivity to entry-activating proteolysis offer plausible explanations for Omicron dominance in human nasal infection and transmission. The findings offer insights into the evolution and prevalence of Omicron viruses and may help define the character of future human emergent VOCs.

## MATERIALS AND METHODS

### Cell lines

HEK293T (obtained from Dr. Ed Campbell, Loyola University Chicago) and Vero-E6-hACE2-hTMPRSS2 (obtained from Dr. Stanley Perlman, University of Iowa) cells were maintained in Dulbecco’s modified Eagle media (DMEM)-10% fetal bovine serum (FBS) (DMEM containing 10 mM HEPES, 100 nM sodium pyruvate, 0.1 mM non-essential amino acids, 100 U/mL penicillin G, and 100 µg/mL streptomycin, and supplemented with 10% FBS; Atlanta Biologicals). All cell lines were cultured in a 5% CO_2_ incubator at 37°C.

### Plasmid construction

Full-length SARS-CoV-2 S, E, M, and N genes (GenBank: NC_045512.2) were synthesized by Genscript, Inc. as human codon-optimized cDNAs and inserted into pcDNA3.1 expression vectors. Omicron BA.1 S was synthesized (Integrated DNA Technologies). All S recombinants were constructed via gene fragment Assembly (NEB, Ipswich, MA USA; #E2621S). HiBiT-N was constructed by fusing HiBiT peptide (VSGWRLFKKIS) and linker (GSSGGSSG) coding sequences to the 5′ end of the N gene, as described in references (47, 133). Nluc-PS9 was constructed by fusing PS9 SARS-CoV-2 RNA packaging sequence to the 3′ end of an Nluc gene, as described in reference (74). The pCMV-LgBiT expression plasmid was purchased from Promega. pcDNA3.1-hACE2-C9 was obtained from Dr. Michael Farzan, Scripps Florida. pcDNA3.1-hACE2-LgBiT was constructed by fusing the coding sequence of LgBiT to the 3′ end of hACE2 gene. pHEF-VSVG-Indiana was constructed previously (134). hACE2-Fc expression plasmid was constructed previously (47, 52).

### Virus-like particles

HiBiT-N-tagged VLPs were produced as described previously (47, 52, 53, 80, 133). Briefly, equimolar amounts of full-length CoV S, E (envelope), M (membrane), and HiBiT-N encoding plasmids were LipoD (SignaGen, Frederick, MD USA; cat: SL100668) transfected into HEK293T cells. SARS-CoV-2 PS9 transducing VLPs were made by co-transfecting Nluc-PS9 along with viral structural genes (S, E, M, and N-HiBiT) (74, 76). To produce spikeless “No S” VLPs, the S expression plasmids were replaced with empty vector plasmids. At 6-h post-transfection, cells were replenished with fresh DMEM-10% FBS. HiBiT-N VLPs were collected in FBS-free DMEM from 24 to 48 h post-transfection. FBS-free DMEM containing HiBiT-N VLPs was clarified by centrifugation (300 × *g*, 4°C, 10 min; 3,000 × *g*, 4°C, 10 min).

To obtain purified viral particles, clarified VLP-containing FBS-free DMEM was subjected to either size-exclusion chromatography (SEC) or density ultracentrifugation. For SEC, samples were first concentrated 100-fold by ultrafiltration (Sigma-Aldrich, Burlington, MA USA; UFC910096) before SEC (Izon Science Limited, Medford, MA USA; qEVoriginal, usage following product instructions). VLPs were eluted from columns into 2× FBS-free DMEM. Peak VLP fractions were identified after lysis of VLPs by adding LgBiT and measuring complemented Nluc in a luminometer. For density ultracentrifugation, samples were laid over 20% (wt/wt) sucrose and spun [SW28, 7,500 rpm, 4°C, 24 h (47, 135)]. The resulting pellet was resuspended in FBS-free DMEM. PS9 transducing VLPs were density purified between 20% and 50% (wt/wt) sucrose cushions (SW28, 25,000 rpm, 4°C, 3 h). For downstream experiments, VLP inputs were normalized based on their Nluc activity upon LgBiT complementation. Samples were stored at −80°C until use.

### Cell-free fusion assay

hACE2-LgBiT EVs were obtained as described previously (47, 52, 53, 80). Briefly, HEK293T target cells were LipoD transfected with pcDNA3.1-hACE2-LgBiT. At 6 h post-transfection, transfection media were removed, rinsed, and replaced with FBS-free DMEM. Media were collected at 48 h post-transfection, clarified (300 × *g*, 4°C, 10 min; 3,000 × *g*, 4°C, 10 min), and concentrated 100-fold by ultrafiltration (Sigma-Aldrich, Burlington, MA USA; UFC910096). EVs were then purified using SEC (Izon Science Limited, Medford, MA, USA; qEVoriginal) using PBS pH 7.4 as eluant. Peak EV fractions were identified by adding HiBiT-containing detergent and subsequent Nluc measurement by luminometry. EVs were stored at 4°C.

Cell-free fusion assays were performed as described previously (47, 52, 53, 80). Briefly, at 4°C, HiBiT-N VLPs and hACE2-LgBiT EVs were mixed with nanoluc substrate (Promega, Madison, WI USA; #N2420), DrkBiT [10 µM, peptide sequence VSGWALFKKIS (136), synthesized by Genscript], and trypsin (Sigma-Aldrich, Burlington, MA USA; #T1426; 1 ng/µL or as indicated) in 384-well multiwell plates. Sample plates were then loaded into a Glomax luminometer maintained at 37°C. Nluc accumulations were recorded over time. VLP-EV cell-free fusions were quantified as the fold increase of Nluc signal from S-bearing VLPs over the signal from spikeless (No S) VLP background control.

For hACE2-Fc [made in-house (47, 52)] neutralization, VLPs were incubated with serial dilutions of hACE2-Fc for 30 min at 37°C before adding hACE2-LgBiT EVs, substrate, DrkBiT, and trypsin. For HR2 peptide neutralization, VLPs were incubated with serial dilutions of SARS-CoV-2 HR2-cholesterol [gift from Matteo Porotto and Anne Moscona, Columbia University (34, 53)] for 30 min at 37°C before adding hACE2-LgBiT EVs, substrate, DrkBiT, and trypsin. For trypsin activation, VLPs were incubated with hACE2-LgBiT EVs for 30 min at 37°C before adding substrate, DrkBiT, and serial dilutions of trypsin.

### Pseudoviral particles

VSVGΔG-fluc-G pseudoviral particles [VSVpps, reference (137)] stock was made as described in reference (135). Briefly, HEK293T cells were transfected with VSVG. The next day, seed VSVΔG-G particles were inoculated onto the transfected cells for 2 h. The cells were rinsed two times with FBS-free DMEM medium and replenished with DMEM-10% FBS. Media were collected and clarified (300 × *g*, 4°C, 10 min; 3,000 × *g*, 4°C, 10 min) after a 48-h incubation period.

### Cell transduction

SARS-CoV-2 Nluc-PS9 VLPs and VSVpps were used to transduce Vero-E6-hACE2-hTMPRSS2 cells. For hACE2-Fc neutralization, particles were incubated with serial dilutions of hACE2-Fc for 30 min at 37°C before inoculating onto cells. For HR2 neutralization, VLPs were incubated with serial dilutions of SARS-CoV-2 HR2-cholesterol for 30 min at 37°C before inoculating onto cells. For hTMPRSS2 inhibition, cells were incubated with titrated levels of camostat (Sigma-Aldrich, Burlington, MA USA; #SML0057) for 30 min at 37°C before VLPs were inoculated. Inoculation was allowed for 2 h, then cells were rinsed three times and replenished with DMEM-10% FBS. After 4 h, the cells were lysed by Passive lysis buffer (Promega, Madison, WI USA; #E1941) or lysis buffer [25  mM Tris-phosphate (pH 7.8), 2  mM dithiothreitol (DTT), 2  mM 1,2-diaminocyclohexane-*N*,*N*,*N*′-tetraacetic acid, 10% (vol/vol) glycerol, and 1% Triton X-100], for VLPs and pp inoculations, respectively. Nluc (VLP) activity was recorded after substrate (Promega, Madison, WI USA; #N1110) addition, and Fluc (VSVpp) activity was recorded after substrate [1  mM d-luciferin, 3  mM ATP, 15  mM MgSO_4_·H_2_O, 30  mM HEPES (pH 7.8)] addition, by a Veritas microplate luminometer.

### Western blot

Samples in SDS solubilizer (0.0625 M Tris·HCl [pH 6.8], 10% glycerol, 0.01% bromophenol blue, 2% (wt/vol) SDS, ±2% 2-mercaptoethanol) were heated at 95°C for 5 min, electrophoresed through 8% or 10% (wt/vol) polyacrylamide-SDS gels, transferred to nitrocellulose membranes (Bio-Rad, Hercules, CA USA), and incubated with mouse monoclonal anti-SARS-CoV-2-S1 (R&D Systems, Minneapolis, MN USA; cat: MAB105403), mouse monoclonal anti-SARS-S2 (Thermo Fisher Scientific, Waltham, MA USA; cat: MA5-35946), mouse monoclonal anti-β-actin-peroxide (Sigma-Aldrich, Burlington, MA USA; cat: A3854), or purified LgBiT-substrate cocktail (Promega, Madison, WI USA; cat: N2420). After incubation with appropriate HRP-tagged secondary antibodies and chemiluminescent substrate (Thermo Fisher Scientific, Waltham, MA, USA; cat: PI32106), the blots were imaged and processed with a FlourChem E (Protein Simple, San Jose, CA, USA).

### Quantification and statistical analysis

All titration curves are one representative of at least three biological repeats. For these graphs, mean and SD are shown based on three technical replicates. To quantitatively compare the effects of spike mutations on S functions, the IC50 or EC50 values from each biological replicate were pooled and subsequently normalized to the parental D614G S control, whose IC50 or EC50 values were set to 1.0. Mean and SEM are shown based on data from biological repeats. Values from all conditions were tested for their deviation from the reference value of 1.0 (D614G parent), and their statistical significances were determined using one-sample *t* tests. All graphs and statistical analyses were completed using Prism 8 (GraphPad, La Jolla, CA USA). *P* values less than 0.05 were considered statistically significant.

## SUPPLEMENTAL MATERIAL

Figure S1 mbio.00171-23-s0001.eps Effects of VLP S protein levels and VLP S protein cleavage status on VLP-EV fusion measurements. Panels A-D: The levels of recombinant S proteins in VLPs (Sunc + S2) were quantified from the western blot band intensities in Fig. 4 and plotted as S / N protein ratios; D614G S / N was set to 1.0. The variations in S protein levels were plotted relative to the VLP-EV fusion measurements recorded in Figs. 4-7. These include maximum VLP-EV fusion levels (Panel A), hACE2-Fc neutralization sensitivities (Panel B), HR2 peptide neutralization sensitivities (Panel C), and trypsin activation of fusion (Panel D). Pearson coefficient (R^2^) and probability (P) values are shown. R2 > 0.7 and P < 0.05 values are taken to indicate statistically significant correlations. The only statistically significant correlation was between S protein levels and maximum VLP-EV fusion.

Figure S2 mbio.00171-23-s0002.eps Effects of VLP S protein cleavage extents on VLP-EV fusion measurements. Panels A-D: The extents of S protein cleavage in VLPs (S2 / Sunc + S2) were quantified from western blot band intensities in Fig. 4 and plotted as relative S1/S2 cleavage; D614G S cleavage extent was set to 1.0. The variations in S1/S2 cleavage were plotted relative to the VLP-EV fusion measurements recorded in Figs. 4-7. These include maximum VLP-EV fusion levels (Panel A), hACE2-Fc neutralization sensitivities (Panel B), HR2 peptide neutralization sensitivities (Panel C), and trypsin activation of fusion (Panel D). Pearson coefficient (R^2^) and probability (P) values are shown. R2 > 0.7 and P < 0.05 values are taken to indicate statistically significant correlations. There were no statistically significant correlations between S1/S2 cleavage status and VLP-EV fusion measurements.

Figure S3 mbio.00171-23-s0003.eps Omicron BA.1 S2 adaptations. SARS-CoV-2 S prefusion (panels A and B; PDB: 7KRR) and postfusion (panels C and D; PDB: 6XRA) structures. NTD is colored in red, RBD in green, SD1&2 in blue, and S2 in orange. Changes from ancestral D614G to omicron BA.1 are labeled and colored in magenta. Panel A and C, S trimer. Panels B and D, a single S protomer. Omicron-adapted residues found to affect receptor reactivity are colored in blue. Residues not visible on the structures are noted with asterisks.

ASM does not own the copyrights to Supplemental Material that may be linked to, or accessed through, an article. The authors have granted ASM a non-exclusive, world-wide license to publish the Supplemental Material files. Please contact the corresponding author directly for reuse.
